# Effects of Sleep-Extend on glucose metabolism in women with a history of gestational diabetes: a pilot randomized trial

**DOI:** 10.1186/s40814-022-01076-2

**Published:** 2022-06-04

**Authors:** Sirimon Reutrakul, Pamela Martyn-Nemeth, Lauretta Quinn, Brett Rydzon, Medha Priyadarshini, Kirstie K. Danielson, Kelly G. Baron, Jennifer Duffecy

**Affiliations:** 1 Division of Endocrinology, Diabetes and Metabolism, Department of Medicine, University of Illinois at Chicago, 835 S. Wolcott, Suite E625, Chicago, IL 60612 USA; 2 Department of Biobehavioral Nursing Science, College of Nursing, University of Illinois at Chicago, 845 S. Damen, Chicago, IL 60612 USA; 3 Division of Public Health, Department of Family and Preventive Medicine, The University of Utah, 375 Chipeta Way, Salt Lake City, USA; 4 Departments of Psychology and Psychiatry, The University of Utah, 501 Chipeta Way, Salt Lake City, UT 84108 USA; 5 Department of Psychiatry, University of Illinois at Chicago, 912 S. Wood Street, Chicago, IL 60612 USA

**Keywords:** Gestational diabetes, Sleep extension, Glucose, Sleep duration, Short sleep

## Abstract

**Objectives:**

Women with a history of gestational diabetes (GDM) are at 7-fold increase in the risk of developing diabetes. Insufficient sleep has also been shown to increase diabetes risk. This study aimed to explore the feasibility of a sleep extension in women with a history of GDM and short sleep, and effects on glucose metabolism.

**Methods:**

Women age 18–45 years with a history of GDM and actigraphy confirmed short sleep duration (<7 h/night) on weekdays were randomized at a ratio of 1 control (heathy living information) to 2 cases (6 weeks of “Sleep-Extend” intervention: use of a Fitbit, weekly digital content, and weekly coaching to increase sleep duration). An oral glucose tolerance test (OGTT), 7-day actigraphy recording, and questionnaires were obtained at baseline and 6 weeks. Mean differences between baseline and end-of-intervention parameters were compared using independent samples *t*-tests.

**Results:**

Mean (SD) sleep duration increased within the Sleep-Extend group (*n*=9, +26.9 (42.5) min) but decreased within the controls (*n*=5, − 9.1 (20.4) min), a mean difference (MD) of 35.9 min (95% confidence interval (CI) − 8.6, 80.5). Fasting glucose increased, but less in Sleep-Extend vs. control groups (1.6 (9.4) vs 10.4 (8.2) mg/dL, MD − 8.8 mg/dL (95% CI − 19.8, 2.1), while 2-h glucose levels after an OGTT did not differ. Compared to controls, Sleep-Extend had decreased fatigue score (MD − 10.6, 95%CI − 20.7, − 0.6), and increased self-report physical activity (MD 5036 MET- minutes/week, 95%CI 343, 9729. Fitbit compliance and satisfaction in Sleep-Extend group was high.

**Conclusion:**

Sleep extension is feasible in women with a history of GDM, with benefits in fatigue and physical activity, and possibly glucose metabolism. These data support a larger study exploring benefits of sleep extension on glucose metabolism in these high-risk women.

**Trial registration:**

ClinicalTrials.gov, NCT03638102 (8/20/2018)

## **Key messages regarding feasibility**

1. Uncertainties existed whether sleep extension in women with a history of gestational diabetes and insufficient sleep is able to increase sleep duration, and if this will improve glucose metabolism.
2. Key feasibility findings are that Sleep-Extend was well received and able to increase nightly sleep duration, with possible favorable effects on fasting glucose levels.
3. These data support a design of a larger randomized trial exploring the effects of Sleep-Extend on glucose metabolism in these women at high risk of future diabetes.

## Introduction

Gestational diabetes mellitus (GDM) affects 4–9% of pregnant women [1]. Women with a history of GDM are at a 7-fold higher risk of developing future type 2 diabetes compared to those with normoglycemia during pregnancy [2]. The public health impact of GDM is significant as it was estimated that 10–31% of parous women with type 2 diabetes had a prior history of GDM [3]. Thus, reducing future diabetes risk in women with a history of GDM who have not yet developed diabetes is crucial to reduce future type 2 diabetes burden [4].

While there are well-established risk factors for type 2 diabetes development, including obesity, physical inactivity, and family history of diabetes, insufficient sleep (i.e., <7 h/night) is increasingly recognized as an independent risk factor [5]. Experimental sleep restrictions in healthy volunteers have shown that sleeping between 4 and 5.5 h for 1–14 nights led to 16–25% reduction in insulin sensitivity [6–9]. Prospective observational studies revealed that insufficient sleep is a risk factor of incident diabetes [10–12]. Sleeping ≤5 h was associated with a 48% increase in the risk of developing diabetes in a meta-analysis of more than 580,000 participants, while sleeping 6 h was associated with an 18% increase [5].

Poor sleep was reported to be associated with altered glucose metabolism in women with a history of GDM. For example, poorer self-reported sleep quality was associated with higher 2-h glucose levels post 75-g oral glucose tolerance test (OGTT) [13], and sleep duration ≤6 h was associated with significantly higher fasting,1 h, and area under the curve of glucose levels post OGTT than sleeping >6 h [14]. Thus, these data strongly support that sleep plays a role in glucose metabolism in women with previous GDM, and represents a potentially modifiable risk factor in reducing future diabetes risk.

Despite strong data to support a causal relationship between insufficient sleep and abnormal glucose metabolism derived from experimental and observational studies, only a few emerging published studies have explored sleep interventions to improve metabolic outcomes. One study demonstrated that sleep extension in chronic short sleepers for 6 weeks resulted in an average 44 min increase in nightly sleep duration, and there was a correlation between the increase in sleep duration and improvement in fasting insulin sensitivity [15]. In another randomized cross-over sleep extension study of 21 short-sleeping non-diabetic working-age adults, those who extended their sleep to >6 h/night for 2 weeks had significant improvement in fasting insulin resistance (HOMA-IR), early insulin response to glucose, and β-cell function [16]. However, another 4-week randomized controlled trial (RCT) of 42 normal-weight, healthy, short sleepers showed that there were no significant changes in glycemic parameters despite an increase in sleep duration of 32 min/night [17]. Furthermore, there were variations in the ability of participants to increase their sleep duration in these studies. One recent meta-analysis including three studies did not find differences in fasting glucose levels or insulin resistance between control and sleep extension groups [18]. Overall, these results were mixed and no studies specifically examined women with a history of GDM.

Therefore, the aim of this pilot study was to explore the feasibility (e.g., acceptability and choice of appropriate outcomes of the future trial) of technology-assisted behavioral sleep extension “Sleep-Extend” in women with a history of GDM, and its preliminary effects on patient-centered outcomes including sleep duration and glucose metabolism parameters. We hypothesized that Sleep-Extend would be feasible and well-accepted among the participants and able to increase sleep duration and improve glucose metabolism.

## Participants and methods

The aim of the study was to explore the effects of Sleep-Extend, compared to healthy living control, on sleep and glucose metabolism in women with a history of GDM and insufficient sleep, using a randomized controlled trial. The study was conducted at the University of Illinois at Chicago, Chicago, Illinois.

Premenopausal women, age 18–45, with a history of GDM were recruited. Inclusion criteria were being at least one year post-partum, reported habitual sleep duration <7h/night during work- or weekdays with a desire to sleep longer (confirmed by one-week actigraphy), reported time in bed ≤8 h/night, own a smartphone compatible with Fitbit, no need to provide care at night for child (ren) (>3 times/week and > 30 min/time), no history of sleep disorders, and no use of sleep aids. Exclusion criteria were hemoglobin A1C≥6.5% at screening, pregnancy or breast feeding, insomnia symptoms, shift work, high risk for sleep apnea (STOP BANG questionnaire) [19], and significant medical comorbidities. Recruitment was done through emails, advertising in a research website, recruitment letters to patients previously seen at the University of Illinois at Chicago and flyers posted in local clinics. Interested participants were contacted and phone screening was performed by investigators or research assistant. Those who passed phone screening had their in-person visit at the Clinical Research Center at the University of Illinois at Chicago where the informed consent was obtained by investigators or a research assistant. Hemoglobin A1C and pregnancy tests were then performed and those eligible then proceeded in the protocol. The study was approved by the Institutional Review Board, University of Illinois at Chicago (protocol 2018-0092), and registered at ClinicalTrails.gov (NCT03638102, https://clinicaltrials.gov/ct2/show/NCT03638102). The study period was from February 2019 to July 2021 and performed at the University of Illinois at Chicago.

### Assessments

At baseline, weight (kg) and height (cm) were measured. The participants were asked about the number of children living at home. Feasibility, primary and secondary outcomes of interests were assessed at baseline and end of interventions as below.

Feasibility and acceptability were assessed using participants’ feedback to Sleep-Extend (satisfaction score 1-7 and open feedback) and the number of days of Fitbit® use during the intervention in the Sleep-Extend arm. Choices of appropriate outcomes in the future trial were chosen if there was a trend of a difference (statistically or clinically) between the two groups.

The first primary outcome of interest was mean objective sleep duration obtained using Actiwatch 2 (Philips Respironic, Bend, Oregon) for one week. Bedtime and wake time were set by the researcher using the event markers, a sleep log data as well as an in-person review of sleep timing with the participants upon return of the watch. Sleep duration was defined as the amount of actual sleep obtained at night. Sleep efficiency was the percentage of time in bed spent sleeping. Both parameters were calculated using Actiware 6.0 software, supplied by the manufacturer.

The second primary outcomes of interest included fasting glucose level and glycemic parameters obtained from a 75-g oral glucose tolerance test (OGTT). At the end of each sleep period, after an overnight fast, 75-g glucose was given orally. Blood samples were obtained at 0, 30, 60, 90, and 120 min for glucose, and insulin was obtained at 0 min. Glucose levels were assayed by Quest Diagnostics. Serum insulin was measured by ELISA (Mercodia, Winston Salem, NC) or Radioimmunoassay (Immulite 2000). Area under the curve (AUC) of glucose was calculated using the trapezoid rule. Homeostatic Model Assessment of Insulin Resistance (HOMA-IR), an index of fasting insulin resistance, was calculated [20].

The rationale to have two different primary outcomes was due to this being the pilot study which will help plan a future larger study. We needed to demonstrate that the intervention could increase sleep duration. Glucose metabolism parameters are very important metabolic health-related outcomes in this group of participants. The two outcomes were analyzed independently and the relationship between the two was explored (see the “Statistical analysis” section).

Secondary outcomes included weight and self-reported measures assessed by standardized questionnaires. Self-reported sleep quality in the past month was assessed using the Pittsburgh Sleep Quality Index (PSQI), with a higher score reflecting poorer sleep quality [21]. Anxiety symptoms were assessed by General Anxiety Disorder-7 (GAD-7), with a higher score reflecting more anxiety [22]. Fatigue was assessed using the PROMIS Fatigue [23], and self-reported physical activity was assessed using the International Physical Activity Questionnaire (IPAQ)) [24]. Lastly, depressive symptoms were assessed by the Center for Epidemiologic Studies Depression Scale (CES-D) [25].

### Randomization and blinding

Participants were randomized using computer-generated sequence to Sleep-Extend or Healthy living control group at a ratio of 2:1. This ratio was used as a previous sleep extension study revealed that some participants could not adequately extend their sleep [16], thus having more participants in the intervention group would allow us to gain more insight on the effects of the intervention. Randomization was performed by the investigators at the start of the intervention and there was no concealment. Due to the nature of the interventions, participants and investigators were not blinded.

### Sleep-Extend

The goal of Sleep-Extend was to increase sleep time by ≥30 min. Participants received: (1) a wearable sleep tracker (Fitbit®) which they wore during the intervention. The coach had access to the data which was used to guide the coaching. This was chosen as the sleep data could be seen in real time by the coach and it was not practical to have the participants wear an Actiwatch 2 and come back for a download weekly. Fitbit® was shown to provide similar estimates of sleep outside the laboratory to a research grade actigraph [26];(2) the Fitbit smartphone application with interactive feedback and tools; (3) Didactic content (2–3 pages, 5–10 min reading) delivered weekly by email (e.g., education about healthy sleep); (4) Weekly brief telephone coaching sessions (5–20 min) where the coach reinforced the didactic content, provided feedback to the participant based on wearable sleep tracker data, reviewed progress, problem solved barriers, and set goals for the following week. Participants received the Fitbit® and monetary compensation.

### Healthy living control

Participants assigned to the control group were provided weekly health education emails (e.g., vaccination for adults, cancer screening) and received weekly brief telephone contact from the coach (≤ 5 min) to answer questions. They received similar compensation as the Sleep-Extend group along with Fitbit® at the end of the protocol.

### Statistical analysis

As this was a pilot and feasibility study, the analyses were exploratory. We quantified effect sizes of outcomes between Sleep-Extend and healthy living control (i.e., means, standard deviations (SDs)) to inform the sample size calculations of planned future larger study. Data were expressed as mean (SD) or median (interquartile range, IQR) for continuous variables or frequency (%) for categorical variables. Differences between groups (baseline characteristics and changes between baseline and end of intervention) were compared using independent samples t-tests or Mann-Whitney U tests as appropriate. Correlations between changes in sleep duration, glycemic parameters and questionnaire scores were analyzed using Pearson’s correlation. A *P*-value of < 0.05 (two-sided) was considered statistically significant. Note that the results were preliminary and should be treated with caution in this small study.

## Results

**Fig. 1:**
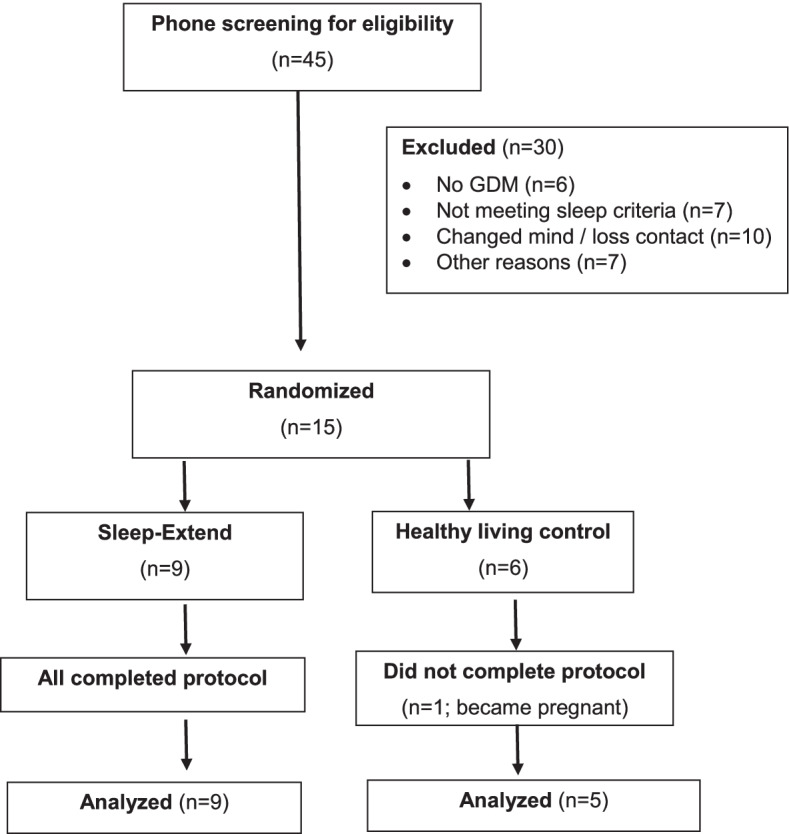
Flow of the study

The consort diagram is shown in Fig. 1. Of the 45 participants screened for eligibility, 15 agreed to the study and randomized (9 Sleep-Extend and 6 controls). One control participant became pregnant after receiving all interventions and did not return for the repeated assessment, resulting in 14 participants completing the study (9 Sleep-Extend and 5 controls). Baseline characteristics are shown in Table 1. Mean fasting glucose for the sample combined was 100.2 (10.8) mg/dL reflecting prediabetes status with no difference between groups. The two groups were comparable in their baseline age, body mass index, number of children living at home, and PSQI score. The Sleep-Extend group, however, had shorter baseline sleep duration (*p*=0.024), more anxiety (*p*=0.035) and depressive symptoms (*p*=0.002), and a trend towards more fatigue than the control group.

**Table 1:** Baseline characteristics of participants

	Sleep-Extend (***n***=9)	Healthy living (***n***=5)	Mean difference	95% confidence interval
***Demographics and self-reported questionnaires***				
Age (years)	42.0 (2.9)	38.7 (6.0)	3.3	− 1.8, 8.4
Number of children living at home	2.2 (0.9)	1.4 (1.3)	0.8	− 0.5, 2.2
Weight (kg)	85.7 (14.3)	85.7 (13.5)	− 0.01	− 17.1, 17.0
BMI (kg/m^2^)	32.7 (5.3)	32.6 (4.6)	0.2	− 5.9, 6.3
PSQI	7.8 (2.9)	6.4 (3.0)	1.4	− 2.2, 4.9
GAD-7 score	3.8 (2.3)	0.8 (1.5), *n*=4	3.0	0.3, 5.8
Promis Fatigue *T*-score	52.3 (8.8)	41.7 (6.3), *n*=4	10.6	− 0.3, 21.3
IPAQ (MET- minutes/week)	3051 (1945, 3723)	8397 (885, 9252)		*U*=18.0, *z*= − 0.60
CES-D	8.9 (3.1)	2.8 (2.2)	6.1	2.6, 9.6
***Objectively measured sleep parameters***				
Sleep duration (minutes)	369.6 (30.8)	408.9 (18.3)	− 39.3	− 72.4, − 6.1
Sleep efficiency (%)	88.2 (3.2)	90.7 (2.5)	− 2.5	− 6.1, 1.2
***Glycemic parameters***				
Fasting glucose (mg/dL)	101.0 (94.0, 106.0)	97.0 (93.0, 100.0)		*U*=14.0, *z*= − 1.14
2h glucose (mg/dL)	144.2 (29.9)	136.0 (25.1)	8.2	− 26.4, 42.8
AUC glucose	304.4 (58.8)	295.4 (26.5)	9.0	− 52.2, 70.3
HOMA-IR*	3.6 (2.1, 4.4)	1.5 (1.2, 3.9)		*U*=11.0, *z*=− 1.32

Data are presented as mean (SD) or median (IQR). *U* and *z* values are from Mann-Whitney *U* tests

*AUC* area under the curve, *BMI* body mass index, *CES-D* Center for Epidemiologic Studies Depression Scale, *GAD-7* General Anxiety Disorder-7, *HOMA-IR* Homeostatic Model Assessment for Insulin Resistance, *IPAQ* International Physical Activity Questionnaire, *PSQI*Pittsburgh Sleep Quality Index

*n=8

**Fig. 2:**
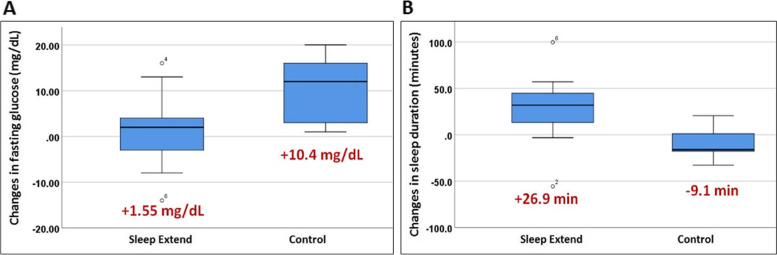
Changes in fasting glucose (**A**) and sleep duration (**B**) in Sleep-Extend and healthy living control groups

Changes after each intervention are shown in Table 2 and Fig. 2. At the end of the protocol, the Sleep-Extend group had increased sleep duration by 26.9 (42.5) min while the control group decreased by 9.1 (20.4) min, with a mean difference (MD) of 35.9 min (95% confidence interval (CI) − 8.6, 80.5), *p*=0.105, Fig. 2. Fasting glucose increased in both groups but less in Sleep-Extend vs. control groups (1.6 (9.4) mg/dL vs 10.4 (8.2) mg/dL, MD − 8.8 mg/dL (95% CI − 19.8, 2.1), *p*=0.103), Fig. 2. Changes in other glycemic parameters did not differ between groups. Compared to controls, Sleep-Extend had decreased fatigue score (MD − 10.6, 95%CI − 20.7, − 0.6), *p*=0.040) and increased self-report physical activity (MD 5036 MET- minutes/week, 95%CI 343, 9729, *p*=0.038). Other outcomes including PSQI, GAD-7, CES-D, and weight did not differ between groups.

**Table 2:** Changes in sleep, glycemic, and self-reported parameters after interventions

	Sleep-Extend (***n***=9)	Healthy living (***n***=5)	Mean difference	95% confidence interval
***Objectively measured sleep parameters***				
Δ sleep duration (minutes)	26.9 (42.5)	− 9.1 (20.4)	35.9	− 8.6, 80.5
Δ sleep efficiency (%)	− 3.0 (3.1)	− 0.6 (1.9)	− 2.4	− 5.7, 0.9
Δ fasting glucose (mg/dL)	1.6 (9.4)	10.4 (8.2)	− 8.8	− 19.8, 2.1
Δ 2h glucose (mg/dL)	8.7 (20.6)	10.4 (27.3)	− 1.6	− 29.6, 26.4
Δ AUC glucose	13.5 (35.1)	6.6 (46.7)	6.9	− 40.9, 54.8
Δ HOMA-IR	0.70 (1.40)*	0.59 (1.08)	0.11	− 1.51, 1.74
***Secondary outcomes***				
Δ weight (kg)	− 0.22 (1.9)	− 1.66 (2.08)	1.43	− 0.97, 3.85
Δ PSQI	− 2.8 (3.2)	− 2.0 (4.2)	− 0.8	− 5.1, 3.5
Δ GAD-7 score	1.0 (− 1.5, 2.0)	1.0 (0.0, 2.0)**		*U*=17.0, *z*=− 0.16
Δ Promis fatigue *T*-score	− 5.1 (8.7)	5.5 (3.1)**	− 10.6	− 20.7, − 0.6
Δ IPAQ (MET- minutes/week)	+1847 (3200)	− 3188 (4341)**	5036	343, 9729
Δ CES-D	− 1.9 (5.6)	+3.4 (3.8)	− 5.2	-11.4, 0.9

Data are presented as mean (SD) or median (IQR). *U* and *z* values are from Mann-Whitney *U* tests

*AUC* area under the curve, *BMI* body mass index, *CES-D* Center for Epidemiologic Studies Depression Scale, *GAD-7* General Anxiety Disorder-7, *HOMA-IR* Homeostatic Model Assessment for Insulin Resistance, *IPAQ* International Physical Activity Questionnaire, *PSQI* Pittsburgh Sleep Quality Index

*n=8, **n=4

When analyzing all participants, there was a moderate but non-significant correlation between an increase in sleep duration and both: a decrease in fasting glucose (*r* = − 0.448, *p*=0.108); and a decrease in fatigue score (*r*= − 0.518, *p*=0.070, *n*=13). In Sleep-Extend, those with shorter baseline sleep duration tended to have a larger increase in sleep duration after the intervention (*r* =− 0.591, *p*=0.094)

For feasibility, eight of nine Sleep-Extend participants wore Fitbit 100% of the study days. The satisfaction rate was high (score 6.56/7) and there were overwhelming positive feedbacks of the coaching (e.g., *helpful and encouraging coach, helpful tips and helped me realize what I was doing wrong and how to improve, talking to coach every week pushed me to make changes*). According to the results above, sleep duration, glucose levels, fatigue and physical activities should be outcomes explored in a future trial.

Of note, due to COVID-19 and closure of non-therapeutic research and participant’s schedule, there was a delay in the assessment at the end of the protocol in 4 participants (3 Sleep-Extend and 1 control), occurring 12 weeks (*n*=2) and 20 weeks (*n*=2) after the start of the protocol. One subject had pain and numbness at the blood draw site that resolved within a few days without treatment. There were no other adverse events.

## Discussion

In this study, we demonstrated that a technology-assisted behavioral sleep extension was feasible and well received among women with GDM history and short sleep as indicated by high rate of Fitbit use and satisfaction. Although not statistically significant, likely due to small number of subjects in this pilot study, there was a trend in increased sleep duration after the intervention (MD of 36 more minutes compared to controls) and a favorable trend in fasting glucose levels (a difference of 8.8 mg/dL lower compared to controls) which is clinically significant, especially in this group of women who are at high risk of developing diabetes. There was an improvement in self-reported fatigue and physical activity in Sleep-Extend. These outcomes of interests should be confirmed in a larger study.

The magnitude of increase in sleep duration during interventions in our study, 36 min, is in agreement with a few previous sleep extension studies, ranging between 32 and 73 min/night [15–17, 27], suggesting that women with a history of GDM responded similarly to other short sleepers. A recent meta-analysis including 26 sleep extension studies in various populations (only a few measured glycemic outcomes) also revealed that an average increase in sleep duration was 48 min [28]. This is especially important as these women have responsibilities (e.g., work, child care) which might cause them to curtail their sleep. The acceptability of the intervention suggested that this suited their lifestyle and schedules, supporting this strategy in planning further study. This increase in sleep duration is also considered significant as evidence suggested a relationship between amount of sleep and glucose levels. For example, in one study of pregnant women, each hour of reported reduced sleep time was associated with a 4% increase in glucose levels after a glucose challenge test [29]. In the current study, an effect size of 8.8 mg/dL in fasting glucose levels from the intervention could be clinically significant as each mg/dL increase in fasting glucose levels was shown to be associated with a 6% increase in the diabetes risk [30]. This effect size needs to be confirmed in a larger study.

Insufficient sleep has been linked to abnormal glucose metabolism via several mechanisms, most of which were derived from experimental studies. These include elevated sympathetic nervous system activity, alterations of cortisol and growth hormone rhythms, alteration of appetite-regulating hormones, elevated inflammatory markers, and adipocyte insulin resistance, leading to increased insulin resistance without adequate insulin secretory response [6–9]. Whether the reversal of these pathologies links to the improvement in glucose metabolism in behavioral sleep extension has not been extensively explored. One study in short sleeping men demonstrated that a 6-week behavioral sleep extension (an increase of approximately 73 min) resulted in lower fasting insulin resistance along with a reduction in fasting peptide YY levels [27]. However, another study in 42 participants showed that sleep extension (4 weeks, average 32 min) did not result in improved glucose metabolism or changes in cortisol, sympathetic tone, leptin, or ghrelin levels, although the sleep extension group reduced intake of fat, carbohydrates, and free sugars in comparison to the control group [17]. One recent study showed the benefit of sleep extension on energy intake and weight reduction, with an inverse correlation between changes in sleep duration and energy intake, although glucose was not an outcome in this study [31]. While a majority of published studies demonstrated favorable effects of behavioral sleep extension on glucose metabolism, these studies were small (16–42 participants), and the mechanisms by which the intervention improves glucose metabolism remain to be explored in detail.

Our study successfully utilized technology-assisted sleep intervention. Many technology interventions suffer from high rates of non-adherence [32]. The use of coached interventions typically shows larger effect sizes than unguided interventions, likely due to improved adherence [33]. The process by which human support enhances adherence to digital interventions has been termed “Supportive Accountability” [34]. This model suggests that participants are more likely to adhere if they are accountable to another person [35]. The model leverages the qualities of the coach, including legitimacy and bond, to improve the use of the intervention. Monitoring adherence and gently holding the participant accountable to their pre-defined goals is at the core of the coaching intervention. We believe that the use of the sleep tracker and remote coaching sessions worked well as proven by participants’ high rate of Fitbit® use and high satisfaction. Increased sleep duration was also clinically significant. Sleep-Extend could potentially be scalable to a wider population as it has been designed to be delivered remotely, highly relevant in the COVID-19 era.

The study has a strength of being the first conducted in women with a history of GDM and short sleep, a group that has an increased risk for diabetes and unique challenges to obtaining adequate sleep, such as balancing work and child care. Limitations included baseline differences (Sleep-Extend group had shorter sleep duration, more anxiety and fatigue than controls), small number of subjects, and protocol interruptions due to COVID-19 (*n*=4). We are designing a larger adequately powered study which can be delivered completely remotely in order to confirm the preliminary findings of this study. Using the mean difference in fasting glucose levels of 8.84 mg/dL (SD=9.7 mg/dL) between the two groups, we calculated that we would need 25 participants per group (after 15% attrition) to provide 90% power with a two-tailed *α*=0.05. In this study, the recruitment was mainly done via electronic advertising, thus participants might have not truly represented the general population with GDM. Further, possible mediators between increased sleep and improved glucose (e.g., inflammatory markers, objectively measured physical activity), as shown in experimental sleep restrictions, should be measured [7, 36]. In this study, those with shorter baseline sleep duration tended to respond better to the intervention, but other moderators should also be explored to better target interventions to specific groups. Since our study was not blinded, there could possibly be bias. However, our primary outcomes were objectively measured and secondary outcomes were assessed by standardized questionnaires in the hope to minimize any possible bias. It also was unclear why fasting glucose levels increased in both groups. While this could be partly due to disease progression, it could possibly be due to other factors not captured by the study.

## Conclusion

This pilot study demonstrated that technology-assisted behavioral sleep extension in women with GDM and short sleep was feasible with a trend in favorable outcomes in both sleep duration, fatigue and physical activity, and possibly for fasting glucose. This will next be confirmed in a larger study.

## Data Availability

The datasets used and/or analyzed during the current study are available from the corresponding author on reasonable request.
